# Supplementary material for: IL-2, IL-6 and chitinase 3-like 2 might predict early relapse activity in multiple sclerosis
Source: PLoS One. 2022 Jun 27;17(6):e0270607. doi: 10.1371/journal.pone.0270607 (PMC9236235; doi:10.1371/journal.pone.0270607)
Supplement: S1 Table — (PDF) [file pone.0270607.s001.pdf]

S1 Table. Relapse rate.

	ARR - year 1		ARR - year 2		Relapse rate - years 1 + 2	
	Median TST	Cut-off	Median TST	Cut-off	Median TST	Cut-off
	P-value	Group in risk	P-value	Group in risk	P-value	Group in risk
IgG calc	n.s.	positive	n.s.	positive	n.s.	positive
OCGB	n.s.	positive	n.s.	negative	n.s.	positive
IgM calc	n.s.	positive	n.s.	negative	n.s.	positive
OCMB	n.s.	negative	n.s.	negative	n.s.	negative
$Index_{IL-2}$	0.0086	≥0.26	n.s.	≥0.26	0.0165	≥0.26
$Index_{IL-6}$	0.0265	≥0.25	n.s.	≥0.25	0.0327	≥0.25
$Index_{IL-10}$	n.s.	≥0.20	n.s.	≥0.14	n.s.	≥0.14
$Index_{CHI3L2}$	n.s.	≥1.79	n.s.	≥1.79	n.s.	≥1.79
pNfH in CSF (pg/ml)	n.s.	≥95.0	n.s.	≥95.0	n.s.	≥95.0
pNfH in serum (pg/ml)	n.s.	<25.5	n.s.	<23.3	0.0406	<23.3
