# Supplementary material for: IL-2, IL-6 and chitinase 3-like 2 might predict early relapse activity in multiple sclerosis
Source: PLoS One. 2022 Jun 27;17(6):e0270607. doi: 10.1371/journal.pone.0270607 (PMC9236235; doi:10.1371/journal.pone.0270607)
Supplement: S2 Table — (PDF) [file pone.0270607.s002.pdf]

S2 Table. Change in EDSS.

	Cut-off	EDSS at T=0		Change in EDSS T=0 vs T=24						
				Group < cut-off		Group ≥ cut-off		Between groups		
		P-value	Group in risk (higher EDSS)	P-value	increase/ decrease	P-value	increase/ decrease	P-value	increase/ decrease	Group in risk
IgG calc	positive	n.s.	-	0.0195	↓	n.s.	-	n.s.	-	-
OCGB	positive	n.s.	-	n.s.	-	0.0043	↓	n.s.	-	-
IgM calc	positive	n.s.	-	0.0019	↓	n.s.	-	n.s.	-	-
OCMB	positive	n.s.	-	0.0333	↓	0.0483	↓	n.s.	-	-
$Index_{IL-2}$	0.34	n.s.	-	0.0491	↓	0.0313	↓	0.0239	↓	≥0.338
$Index_{IL-6}$	0.25	n.s.	-	n.s.	-	0.0153	↓	n.s.	-	-
$Index_{IL-10}$	0.14	n.s.	-	n.s.	-	0.013	↓	n.s.	-	-
$Index_{CHI3L2}$	1.79	n.s.	-	n.s.	-	0.0306	↓	n.s.	-	-
pNfH in CSF (pg/ml)	95.0	n.s.	-	n.s.	-	0.0022	↓	n.s.	-	-
pNfH in serum (pg/ml)	25.5	n.s.	-	0.0327	↓	n.s.	-	n.s.	-	-
