# Supplementary material for: IL-2, IL-6 and chitinase 3-like 2 might predict early relapse activity in multiple sclerosis
Source: PLoS One. 2022 Jun 27;17(6):e0270607. doi: 10.1371/journal.pone.0270607 (PMC9236235; doi:10.1371/journal.pone.0270607)
Supplement: S3 Table — (PDF) [file pone.0270607.s003.pdf]

S3 Table. Six months confirmed EDSS worsening.

	Kaplan-Meier	Cut-off			
			K-M	Cox reg	
	P-value	Group in risk	P-value	Hazard Ratio	P-value
IgG calc	n.s.	negative	-	1.8	n.s.
OCGB	n.s.	N/A	-	N/A	N/A
IgM calc	n.s.	positive	-	2.3	n.s.
OCMB	n.s.	positive	-	1.8	n.s.
$Index_{IL-2}$	n.s.	≥0.26	n.s.	1.3	n.s.
$Index_{IL-6}$	n.s.	<0.25	n.s.	2.2	n.s.
$Index_{IL-10}$	n.s.	<0.15	n.s.	N/A	N/A
$Index_{CHI3L2}$	n.s.	<1.79	n.s.	2.1	n.s.
pNfH in CSF (pg/ml)	n.s.	<95.0	n.s.	3.9	n.s.
pNfH in serum (pg/ml)	n.s.	≥51.9	0.0068	9.2	0.0268
