# Supplementary material for: IL-2, IL-6 and chitinase 3-like 2 might predict early relapse activity in multiple sclerosis
Source: PLoS One. 2022 Jun 27;17(6):e0270607. doi: 10.1371/journal.pone.0270607 (PMC9236235; doi:10.1371/journal.pone.0270607)
Supplement: S4 Table — (PDF) [file pone.0270607.s004.pdf]

S4 Table. Lesion load.

	Wilcoxon TST/ Fisher's ET/ Median TST/ Chi-Square	Cut-off			
		Group in risk	Fisher's ET	Odds Ratio	
	P-value		P-value	Value	Confidence Interval
IgG calc	n.s.	positive	-	1.2	n.s.
OCGB	n.s.	N/A	-	N/A	N/A
IgM calc	n.s.	positive	-	1.9	n.s.
OCMB	n.s.	positive	-	1.8	n.s.
$Index_{IL-2}$	n.s.	<0.26	n.s.	1.2	n.s.
$Index_{IL-6}$	n.s.	≥0.25	n.s.	1.7	n.s.
$Index_{IL-10}$	n.s.	<0.13	n.s.	5.0	n.s.
$Index_{CHI3L2}$	n.s.	<1.79	n.s.	1.3	n.s.
pNfH in CSF (pg/ml)	n.s.	<95.0	0.0423	6.5	n.s.
pNfH in serum (pg/ml)	n.s.	≥12.3	n.s.	2.1	n.s.
