# Supplementary material for: IL-2, IL-6 and chitinase 3-like 2 might predict early relapse activity in multiple sclerosis
Source: PLoS One. 2022 Jun 27;17(6):e0270607. doi: 10.1371/journal.pone.0270607 (PMC9236235; doi:10.1371/journal.pone.0270607)
Supplement: S5 Table — (PDF) [file pone.0270607.s005.pdf]

S5 Table. Brain atrophy.

	Wilcoxon TST/ Fisher's ET/ Median TST/ Chi-Square	Cut-off			
		Group in risk	Fisher's ET	Odds Ratio	
	P-value		P-value	Value	Confidence Interval
IgG calc	n.s.	negative	-	1.1	n.s.
OCGB	n.s.	positive	-	N/A	N/A
IgM calc	n.s.	positive	-	1.1	n.s.
OCMB	n.s.	positive	-	1.1	n.s.
$Index_{IL-2}$	n.s.	<0.26	n.s.	3.6	n.s.
$Index_{IL-6}$	n.s.	≥0.25	n.s.	1.1	n.s.
$Index_{IL-10}$	n.s.	<0.13	0.0368	5.7	n.s.
$Index_{CHI3L2}$	n.s.	≥1.79	n.s.	1.1	n.s.
pNfH in CSF (pg/ml)	n.s.	<95.0	n.s.	1.0	n.s.
pNfH in serum (pg/ml)	n.s.	≥10.8	0.0428	9.8	n.s.
