# Supplementary material for: IL-2, IL-6 and chitinase 3-like 2 might predict early relapse activity in multiple sclerosis
Source: PLoS One. 2022 Jun 27;17(6):e0270607. doi: 10.1371/journal.pone.0270607 (PMC9236235; doi:10.1371/journal.pone.0270607)
Supplement: Attachment — Submitted filename: Response to Reviewers.docx [file pone.0270607.s007.docx]

**PONE-D-22-08909**

**IL-2, IL-6 and chitinase 3-like 2 might predict early relapse activity in multiple sclerosis**

Dear Editor, dear Reviewers,

First, we would like to thank you for the time you have spent with our manuscript and for all the comments you have made. We believe that your kind input will surely be reflected in the improved quality of our article. Below, you will find all your comments addressed one by one.

**Journal Requirements:**

When submitting your revision, we need you to address these additional requirements.

1. Please ensure that your manuscript meets PLOS ONE's style requirements, including those for file naming. The PLOS ONE style templates can be found at 
https://journals.plos.org/plosone/s/file?id=wjVg/PLOSOne_formatting_sample_main_body.pdf and https://journals.plos.org/plosone/s/file?id=ba62/PLOSOne_formatting_sample_title_authors_affiliations.pdf

The manuscript and all the submitted files have been rechecked to comply with the journal’s requirements and edited accordingly.

2. Thank you for stating the following in the Competing Interests section: 
"I have read the journal's policy and the authors of this manuscript have the following competing interests: MP received publication honorarium and compensations for travel and conference registration fees from Novartis, Merck Serono and Sanofi Genzyme; all outside the submitted work. EM received speaker honoraria and consultant fees from Novartis, Merck Serono, Sanofi Genzyme, Roche, Biogen Idec and Teva; all outside the submitted work. JL received compensations for travel, speaker honoraria and consultant fees from Novartis, Merck Serono, Sanofi-Genzyme, Roche, Biogen Idec, Teva and Bayer Healthcare; all outside the submitted work. HM received compensations for travel and conference registration fees from Novartis, Merck Serono, Sanofi Genzyme and Roche; all outside the submitted work. ME received publication honorarium, speaker honoraria and compensations for travel and conference registration fees from Novartis, Merck Serono, Roche, Teva and UCB; all outside the submitted work. JH, PR, SK, JŠ, OS, and PM declare that they have no competing interests."

Please confirm that this does not alter your adherence to all PLOS ONE policies on sharing data and materials, by including the following statement: "This does not alter our adherence to  PLOS ONE policies on sharing data and materials.” (as detailed online in our guide for authors <http://journals.plos.org/plosone/s/competing-interests>).  If there are restrictions on sharing of data and/or materials, please state these. Please note that we cannot proceed with consideration of your article until this information has been declared.

Please include your updated Competing Interests statement in your cover letter; we will change the online submission form on your behalf.

The updated Competing Interests statement has been included in the cover letter.

3. Please review your reference list to ensure that it is complete and correct. If you have cited papers that have been retracted, please include the rationale for doing so in the manuscript text, or remove these references and replace them with relevant current references. Any changes to the reference list should be mentioned in the rebuttal letter that accompanies your revised manuscript. If you need to cite a retracted article, indicate the article’s retracted status in the References list and also include a citation and full reference for the retraction notice.

The reference list has been rechecked for any inconsistencies. We used scite_ (available at <https://scite.ai/>) to check for any retracted articles. This tool was able to identify 44 out of 55 references, no retracted articles were found. We checked the rest of the references manually: 1) at the web page of the journal where the article had been published, 2) at <http://retractiondatabase.org/RetractionSearch.aspx>?. No retracted articles were found either. Furthermore, we found some typos in the reference list and few references had to be corrected to comply with the journal’s requirements. One reference has been replaced by a more proper one. Also, reference to our raw data has been included (see responses to Reviewer #2). Below, you will find all the changes made to the reference list; numbers correspond to the revised manuscript (with track changes).

12.	Peakman M, Vergani D. Appendix 2: Major cytokines, cells releasing them, targets and functions. In: Peakman M, Vergani D, editors. Basic and Clinical Immunology. London: Elsevier Health Sciences; 2009. p. 342-4. **edited according to PLOS recommended format**

13.	Schroeter M, Jander S. T-cell cytokines in injury-induced neural damage and repair. Neuromolecular Med. 2005;7(3):183-95. **abbreviation of the journal name corrected**

21.	Stampanoni Bassi M, Iezzi E, Drulovic J, Pekmezovic T, Gilio L, Furlan R, et al. IL-6 in the Cerebrospinal Fluid Signals Disease Activity in Multiple Sclerosis. Front Cell Neurosci. 2020;14:120. **page numbers corrected**

22.	Matsushita T, Tateishi T, Isobe N, Yonekawa T, Yamasaki R, Matsuse D, et al. Characteristic cerebrospinal fluid cytokine/chemokine profiles in neuromyelitis optica, relapsing remitting or primary progressive multiple sclerosis. PLoS One. 2013;8(4):e61835. **page numbers corrected**

23.	Stelmasiak Z, Kozioł-Montewka M, Dobosz B, Rejdak K, Bartosik-Psujek H, Mitosek-Szewczyk K, et al. Interleukin-6 concentration in serum and cerebrospinal fluid in multiple sclerosis patients. Med Sci Monit. 2000;6(6):1104-8. **corrections to year of publication, authors’ names**

24.	Romme Christensen J, Börnsen L, Hesse D, Krakauer M, Sørensen PS, Søndergaard HB, et al. Cellular sources of dysregulated cytokines in relapsing-remitting multiple sclerosis. J Neuroinflammation. 2012;9:215. **page numbers corrected**

33.	Ferreira-Atuesta C, Reyes S, Giovanonni G, Gnanapavan S. The Evolution of Neurofilament Light Chain in Multiple Sclerosis. Front Neurosci. 2021;15:642384. **page numbers corrected**

36.	Teunissen CE, Iacobaeus E, Khademi M, Brundin L, Norgren N, Koel-Simmelink MJA, et al. Combination of CSF N-acetylaspartate and neurofilaments in multiple sclerosis. Neurology. 2009;72(15):1322. **title corrected**

39.	Eikelenboom MJ, Uitdehaag BMJ, Petzold A. Blood and CSF Biomarker Dynamics in Multiple Sclerosis: Implications for Data Interpretation. Mult Scler Int. 2011;2011:823176. **page numbers corrected**

40.	Petržalka M. CSF Biomarkers in early MS; 2022 [cited 2022 March 25]. Database: G-Node [Internet]. Available from: https://doi.org/10.12751/g-node.74jj3f. **new**

41.	Reiber H, Otto M, Trendelenburg C, Wormek A. Reporting Cerebrospinal Fluid Data: Knowledge Base and Interpretation Software. Clin Chem Lab Med. 2001;39(4):324-32. **year and journal title corrected**

45.	The MathWorks I, Natick, Massachusetts, United States. MATLAB and SPM12 Toolbox. Release 2020a [Software]. 2020 [cited 2022 March 25]. **edited according to PLOS recommended format**

46.	Schmidt P. Bayesian inference for structured additive regression models for large-scale problems with applications to medical imaging. Dissertation, LMU München. 2017. Available from: <https://edoc.ub.uni-muenchen.de/20373/1/Schmidt_Paul.pdf>. **edited according to PLOS recommended format**

48.	Beadnall HN, Wang C, Van Hecke W, Ribbens A, Billiet T, Barnett MH. Comparing longitudinal brain atrophy measurement techniques in a real-world multiple sclerosis clinical practice cohort: towards clinical integration? Ther Adv Neurol Disord. 2019;12:1756286418823462-. **cancelled**

48.	Kappos L, De Stefano N, Freedman MS, Cree BA, Radue E-W, Sprenger T, et al. Inclusion of brain volume loss in a revised measure of 'no evidence of disease activity' (NEDA-4) in relapsing-remitting multiple sclerosis. Mult Scler. 2016;22(10):1297-305. **new**

50.	Höfer T, Krichevsky O, Altan-Bonnet G. Competition for IL-2 between Regulatory and Effector T Cells to Chisel Immune Responses. Front Immunol. 2012;3:268. **page numbers corrected**

**Reviewers' comments:**

**Reviewer #1:** Petržalka M et al., have assessed the prognostic value of intrathecal IgM synthesis, cerebrospinal fluid and serum IL-2, IL-6, IL-10, chitinase 3-like 2 and neurofilament heavy chains obtained early after the onset of the disease.

The article could be of interest before that there are following changes that need to be made.
1. Efforts should be made to improve the written style of the article. It is very difficult to follow.

Before the initial submission, the manuscript had been language-edited by a professional language editor, an English native speaker. Despite this fact, to avoid any misunderstandings, we tried to improve the style and rephrase some of the sentences/paragraphs that appeared less clear to us.

2. Graphical representation of the main findings should be done.

We added Fig 2. Relapse free interval - relation to the number of positive biomarkers.

3. It is not clear whether the treated patients were used for the experiments or untreated patients.

The lumbar puncture (and the blood draws) was performed as a diagnostic measure, thus none of the patients were treated at time of the lumbar puncture. Later on, all patients started treatment with DMD. This is stated in the Methods section of the Abstract and in more detail in the Methods section of the main article. The confusion might have been caused by the first sentence of this section, where we state “All treatment naïve patients who started first line treatment at the MS Centre of the Motol University Hospital between January 2017 and May 2018 were considered eligible for the study.”. This is meant to say that we included only patients who started with the first line treatment; those who started directly with the high efficacy therapy were excluded. To further clarify this, we added an explanatory sentence, see line 163.

4. Standard nomenclature should be used. IgM oligoclonal bands (OBM), IgG oligoclonal bands (OBG) are not standard nomenclature. Similarly, Relapse free interval (RFI) is not a standardly used terminology etc.

Instead of OBG, we now use OCGB, instead of OBM, we now use OCMB. As for RFI, we performed a short literature search and found several possible alternatives to it, none of which seemed to be more frequently used than the others: relapse free interval, recurrence free interval, relapse free period, relapse-free survival, disease-free survival, time to first relapse, time to second relapse, time to conversion to clinically definite MS. In an article by Havrdova et al, 2009 (Havrdova E, Galetta S, Hutchinson M, et al. Effect of natalizumab on clinical and radiological disease activity in multiple sclerosis: a retrospective analysis of the Natalizumab Safety and Efficacy in Relapsing-Remitting Multiple Sclerosis (AFFIRM) study. Lancet Neurol. 2009;8(3):254-260. doi:10.1016/S1474-4422(09)70021-3), which is one of the first papers that lead to the establishment of the NEDA concept, no specific terminology or abbreviation is used to address the clinical aspect of NEDA. Therefore, we prefer to continue using “relapse free interval” or “RFI”.

5. There are discordances too. For example oligoclonal bands (OBG) and IgG oligoclonal bands (OBG) have the same short form etc.

This was a typo; in both cases we referred to IgG oligoclonal bands. Corrected accordingly.

**Reviewer #2:** Petržalka et al test the predictive value of various biomarkers for disease progression including IL-2, IL-6, chitinase 3-like 2 and IgM in Serum and CSF in patients with early MS. The content of this study is of value to the community.

There are minor points that should be included into the manuscript:

- the Authors discuss that the function of Chitinase-like proteins are still widely unknown an cite a review from 2011. Due to the growing interest in these proteins as biomarkers in MS, also the literature on their function has grown. Please include some of the recent findings in the introduction (eg. Starossom et al 2019, Cubas-Nunes L et al 2021 and more)

In the section about chitinase proteins, we had aimed to introduce mainly CHI3L2. To provide a better understanding of the involvement of these proteins in MS, we have now included some of the more recent findings, as advised.

- a table describing the changes in clinical parameters (relapse free interval, ARR, change in EDSS, six months confirmed
EDSS worsening) & neuroimaging (lesion load, brain atrophy) during the duration of the study for the entire patient group will be helpful to better capture the characteristics of the patient group, that was studied.

We believe that all the information mentioned above is included in the table with our raw data in a very orderly manner. Although freely available at <https://doi.org/10.12751/g-node.74jj3f>, this table was not mentioned in the manuscript. We have added a reference to it, see line 170. Also, we have added tables describing each measured outcome as Supporting information (see the end of the manuscript).

- The authors discuss that neither of the studied biomarkers proved to have significant predictive value in all of the measured
outcomes. Yet the title of the manuscript suggest exactly thatl. Please change the title to something more neutral/descriptive.

We agree that none of the studied biomarkers proved to have significant predictive value in ALL of the measured outcomes. The title, however, states, that only some of the studied biomarkers (IL-2, IL-6 and chitinase 3-like 2) might predict worse outcome only in some of the measured outcomes (early relapse activity). To further clarify this, we have edited the mentioned sentence in the Discussion, see lines 390-393. To this end, we have also edited some parts of the Conclusion. Thus, we would like to maintain the current title.

- Legend to Figure 1 is missing

Legend has been added.

- several typos in the manuscript should be corrected

Addressed.

- are more stablished Biomarkers such as serum of CSF NfL available for the study group'? If so, these should be included here. If not, please include a brief discussion that this could be helpful also in comparison to the herein studied biomarker.

Neither CSF NfL, nor other biomarkers are available for the study group. A brief discussion was included as advised, see lines 474-477.

Yours Faithfully,

Marko Petržalka and co-authors
